# Criminal Justice Involvement and Young Adult Health: The Role of Adolescent Health Risks and Stress

**Published:** 2019-06

**Authors:** William M. Clemens, Monica A. Longmore, Peggy C. Giordano, Wendy D. Manning

**Affiliations:** 1 Department of Sociology, Bowling Green State University, Bowling Green, OH

**Keywords:** Incarceration, Depression, Stress, Health

## Abstract

**Background:**

Although some studies have found that incarceration is associated with young adults’ poor health, confounding factors including adolescent health risks, and mediating influences such as stress have not been examined in the same study. We assessed whether variation in criminal justice system experience (none, arrest only, incarceration) influenced young adults’ self-reported depressive symptoms and poor physical health after accounting for prospective risks to health including adolescent health risks. We then assessed whether stress mediated associations between criminal justice involvement and the two health indicators.

**Methods:**

Data are from Toledo Adolescent Relationships Study (TARS) (n =990), which included young adults, age 22–29, who have matured during the era characterized by mass incarceration. The dependent variables included a depressive symptoms scale and self-reported poor health. The adolescent health risks included economic disadvantage, body mass index, delinquency, problems with drugs, and prior depressive symptoms. We considered stress as a mediating variable. Sociodemographic characteristics included race/ethnicity, age, and gender. We used ordinary least squares regression and logistic regression analyses. We tested gender, race/ethnicity, and age interactions.

**Results:**

In multivariable models, incarceration, and adolescent health risks (economic disadvantage, prior depression, problems with drugs) were associated with young adults’ depressive symptoms, and stress was a mediating influence. Adolescent delinquency and stress, but not incarceration, were significantly associated with young adults’ self-reported poor health.

**Conclusion:**

This study provided a more nuanced understanding of incarceration and health by accounting for several key confounding factors and testing stress as a mechanism underlying the association. Care for prisoner health during and after incarceration is important for successful reintegration.

## INTRODUCTION

In the U.S. many individuals have come in contact with the criminal justice system. Mass incarceration refers to the reality that a vast population of men and women are confined in federal and state prisons as well as local jails, and an even larger population has experienced arrest.^1^ Mass incarceration, disproportionately affects Black and Hispanic young men.^2^ Ohio is not immune to these trends in criminal justice contact. On average, 224,000 individuals are arrested in Ohio each year and in 2017 the Ohio incarceration population was around 80,000. Much like national trends, incarceration in Ohio disproportionately affects individuals of color.^3^ Although 81% of Ohio’s population is White, around 43% of incarcerated individuals in Ohio are Black and 5% are Hispanic. Thus, the criminal justice system reflects racial and socioeconomic inequality.^4^

The large number of individuals arrested as well as incarcerated in Ohio necessitates understanding the collateral health consequences of criminal justice contact. Early research argued that incarceration may improve health because it provided access to a modicum of health care services.^5^ Yet, more recently, some researchers have concluded that incarceration negatively influenced adult men’s physical and mental health.^6^ Other studies have emphasized that it is important to include criminal activity and sociodemographics when analyzing criminal justice system effects because these background factors may also compromise health outcomes.^7^ However, in a recent study that included men’s and women’s criminal activity and sociodemographic background, incarceration remained a significant predictor of health outcomes.^7,8^ We argue that additional research is needed to explore the underlying mechanism linking incarceration and health outcomes. One compelling explanation for the association between criminal justice contact and health outcomes is that incarceration is a stressor that contributes to deteriorated health post-release.^8^ Yet prior studies have not empirically examined general stress as a mechanism.^9^ Additionally, although most studies have focused on incarceration, some researchers have argued that even minor encounters with police and increased police presence in communities negatively influence health outcomes.^10^ Arrest labels individuals, which may lead to a downward spiral including hindering educational and employment opportunities^11^ that influence health outcomes.^12^

We argue that the association between criminal justice system involvement and the probability of poor physical and mental health may be partially due to prior health risk factors including childhood economic disadvantage.^12^ Economic disadvantage is associated with poor health and arrest in adulthood.^13^ Ross found that economic disadvantage led to greater frequency of depressive symptoms^14^ and others found it to be associated with earlier mortality.^13^ These effects may be especially pronounced for mothers released from prison.^15^ Thus, the effects of early economic disadvantage likely influence physical and mental health and should be incorporated in models examining associations between criminal justice contact and health outcomes.

Adolescent health risks also influence health outcomes during young adulthood and should be considered in studies examining the association between criminal justice contact and health. Along with poverty, adolescents face a number of long-term health risks from their behavior. For example, adolescent delinquency has been associated negatively with health in adulthood.^16^ Perhaps a more persistent health risk behavior is drug use during adolescence. A history of drug problems predicted both diminished cognitive capacity and cardiovascular problems in adulthood.^17^ Similarly, marijuana and cocaine use correlates with adverse mental health outcomes.^18^ Longer drug use careers and poorer health increased the probability that incarcerated drug users experienced unmet health needs.^19^ Adolescent substance abuse is related to both increased chance of incarceration and psychiatric disorders, thus the adverse effects of drug use may be additionally compounded by incarceration.^18^ Finally, some basic indicators of poor health in adolescence are also predictive of poorer health in adulthood. For example, elevated body mass index (BMI) predicted lifelong struggles with obesity and increased odds of earlier mortality.^20^ Likewise, adolescent depression is one of the strongest predictors of later life depression.^21^ As highlighted, numerous high-risk adolescent lifestyle factors and health indicators are linked to decreased health through adulthood.^22^

In summary, there are several gaps in the literature that we attempt to address in the current study. First, although some studies have found that incarceration is associated with young adults’ poor health, confounding factors including adolescent health risks, and mediating influences such as stress have not been examined in the same study. Second, some researchers have argued that even minor encounters including being arrested negatively influence health outcomes. Our study assessed whether criminal justice system experience (none, arrest only, incarceration) influenced young adults’ self-reported health and depressive symptoms after accounting for prospective risks to health including childhood poverty, and adolescent health and lifestyle risks (delinquency, problems with drugs, BMI, earlier depression). We then assessed whether stress mediated associations between criminal justice involvement and health. We based analyses on panel data from an Ohio population-based sample of young adults (age 22–29 years) from the Toledo Adolescent Relationships Study (TARS) (n =990). Incarceration rates in Lucas County, Ohio are lower than the average in Ohio as a whole. Nevertheless, each year approximately 600 individuals are admitted to prison in Lucas County and the prison population has remained around 2000 since 2010.

## METHODS

### Setting and Design

The TARS initially was based on a stratified random sample of seventh, ninth and eleventh graders in the years 2000 and 2001 in Lucas County, Ohio. Census data showed that Lucas County is similar to national statistics regarding income, race, and education.^23^ We collected four subsequent waves of data as individuals transitioned to adulthood, and IRB approval was received for each wave.

### Participants

The baseline sample consisted of 1,321 individuals between age 12 and 18 years. At the fifth interview in 2012 respondents were between age 22 and 29 years. We retained 1,021 individuals for the fifth interview. Respondents completed the survey primarily in their homes using a computer assisted interview procedure (first interview) and on-line (fifth interview). We surveyed primary caregivers, usually mothers, at the first interview separately from adolescents. The TARS drew from school rosters, but respondents did not have to be in school or regularly attend school to be included.^23^ We oversampled Black and Hispanic respondents. We excluded respondents missing on self-reported health (n=6) or depression scale items at the fifth interview (n=2). We also excluded respondents who reported their race as “other” (n=23) because the sample size was too small for multivariable analyses. This resulted in a final analytic sample of 990. In our analytic sample, only two variables had missing values (stress n=1; and problems with drugs n=2). We estimated these missing values in descriptive analyses using single imputation of the data, and multiple imputation with 5 iterations for multivariable analyses. We conducted all analyses using SAS 9.4 in 2018.

### Measures

#### Dependent Variables

##### Depressive Symptoms.

We measured depressive symptoms with an eight-item version of the Center for Epidemiological Studies Depressive Symptoms (CESD) scale.^24^ We asked how often in the last week had respondents felt that each statement was true: (1) “felt depressed”; (2) “everything was an effort”; (3) “felt sad”; (4) “couldn’t get going”; (5) “felt lonely”; (6) “couldn’t shake off the blues”; (7) “trouble sleeping or staying asleep”; and (8) “couldn’t keep focused.” The mean scale ranged from 1 (never) to 8 (every day) (α =0.90). Due to skewness, we log transformed depression in multivariable analyses.

##### Poor Health.

We measured self-reported poor health with the item: “Overall, how would you rate your health?” We dichotomized responses into poor or fair health (11.5%) and not in poor health (excellent/very good/good, 88.5%). Dichotomizing self-reported health has precedence in the literature, and yields similar results to more sophisticated categorical measure of health,^25^ and correlates highly with more specific self-reported and objectively measured conditions.^13^

#### Independent variables

##### Criminal Justice Involvement.

In response to the lack of comprehensive measures of criminal justice system involvement,^26^ we measured incarceration history with a trio of assessments. First, respondents indicated each arrest and whether that resulted in jail time. Second, at each interview respondents provided their residency, and if they selected “in prison,” we coded them as incarcerated. Finally, on the parent questionnaire, we asked caregivers how many times their child “was placed in a juvenile detention facility.” If caregivers responded affirmatively, we coded respondents as incarcerated. We constructed criminal justice involvement with the following mutually exclusive categories: never arrested (63%), arrested only (27%), and incarcerated (9%).

##### Adolescent Health Risks.

We used items from the parent’s questionnaire at the first interview to measure childhood economic disadvantage. Some scholars have recommended that measures of poverty should capture inequality and disadvantage as the dynamic process it is rather than more static measures such as household income or education.^27^ Adapting this strategy, we measured economic disadvantage with an index of items. We summed four dichotomous variables: (1) mother has less than high school education; (2) family ever received public assistance; (3) unemployment is a problem in the neighborhood; and (4) there were times when there was not enough food in the house. This count variable of indicators represents the disadvantaged experiences of respondents during adolescence.^28^

We assessed several adolescent health risks at the first interview including BMI, depression, juvenile delinquency, and problems with drugs. We calculated BMI, which we standardized for juveniles according to CDC age guidelines^29^ and then centered the value in multivariable analyses to give the variable an interpretable zero. In multivariable analyses, we included quadratic BMI to account for non-linearity. We measured juvenile delinquency by asking how often respondents committed each of the following: (1) “steal something less than $50”; (2) “steal something more than $50”; (3) “damage property”; (4) “carry gun”; (5) “attack someone”; (6) “sell drugs”; (7) “break into a building;” and (8) “drunk in a public place.”^30^ Responses ranged from 1 (never) to 9 (daily) (α= 0.75). The mean juvenile delinquency score was 0.29 (range = 1 to 9). We measured problems with drugs by asking, “How often in the past 2 years have you experienced the following because of drug use: (1) “not felt good next day”; (2) “unable to do your best”; (3) “problems with partner”; (4) “hit family member”; (5) “gotten into fights”; (6) “problems with friends”; (7) and “gotten into regrettable sexual situation.”^31^ The scale mean was 1.59, and ranged from 1 (never) to 8 (daily) (α=0. 87). For models predicting depression, we measured early depressive symptoms at baseline, similar to the dependent variable, using the eight-item (CESD) scale^24^ (mean = 2.3, range = 1 to 8) (α=0. 83).

##### Stress.

At the fifth interview, we asked respondents about stress that they experienced due to the following: (1) family members’ health; (2) employment; (3) living arrangements; (4) school; (5) money; (6) romantic relationship; (7) parents; (8) other family members; and (9) friends. The scale mean was 2.08 and the range was 1 (not at all stressed) to 5 (extremely stressed) (α=0.83).

##### Sociodemographic Characteristics.

We controlled for race/ethnicity, which we classified as non-Hispanic White (67.5%), non-Hispanic Black (21.5%), and Hispanic (11%) (measured at the first interview). We also controlled for age at the fifth interview (mean = 25, range = 22 to 29), and self-reported gender (46.1% male and 53.9% female).

### Statistical Analysis

We tested whether arrest or incarceration negatively influenced the health of individuals controlling for adolescent health adverse behavior, childhood economic disadvantage, and stress. First, we tested for differences of means/proportions of each independent variable among those with no criminal justice contact, arrested only, and incarcerated using t-tests and chi-square tests (for categorical variables). We conducted logistic and ordinary least squares regression, respectively, to estimate the effects of arrest and incarceration on depression and physical health. We included incarceration and sociodemographic characteristics (model 1), added childhood disadvantage and adolescent health risks (model 2), and then added stress (model 3) We used the Sobel test to assess whether stress mediated the influence of incarceration on the health outcomes. The Sobel test is a method to determine whether the reduction in the effect of an independent variable (i.e., incarceration) is statistically significant after including a mediating variable (i.e., stress).

## RESULTS

Summarizing the descriptive statistics in Table 1, individuals who had experienced incarceration reported more frequent depressive symptoms compared to individuals who were arrested only, and those who had never been arrested. The frequency of depressive symptoms did not differ significantly among those who experienced arrest only and those with no criminal justice contact. We found that a higher proportion of individuals who were incarcerated, compared with having been arrested only and having no criminal justice contact, reported poorer physical health. Self-reported poor health was not statistically different for those who have and have not been arrested.

On average, individuals who were arrested or incarcerated had increased health risk factors (e.g., prior depression, economic disadvantage, higher BMI, delinquency, problems with drugs) compared to individuals who had never been arrested. Additionally, compared with individuals who have never been arrested, individuals who have been incarcerated, and those who have been arrested only, reported significantly higher delinquency scores. Similarly, individuals who have been incarcerated and those who have been arrested only, reported greater odds of problems with drugs during adolescence, compared to those who have never been arrested. Lastly, those who experienced incarceration exhibited higher general stress than those who experienced arrest only and those with no criminal justice system contact.

### Results for Depressive Symptoms

Table 2 includes the unadjusted and multivariable OLS regressions of log transformed depression on criminal justice system involvement, adolescent health risks, stress, and sociodemographic characteristics. The bivariate (unadjusted) analyses indicated that being arrested, and being incarcerated, compared to never having been arrested, are associated with more frequent depressive symptoms. All of the adolescent health risks, stress, and identifying as Black compared to White were associated with greater frequency of depressive symptoms. In model 1, having experienced incarceration, compared to having never been arrested, was associated with greater frequency of depressive symptoms net of sociodemographic characteristics. Identifying as Black compared to White, and women compared with men reported greater frequency of depressive symptoms.

Model 2 demonstrated that with the inclusion of adolescent health risks and sociodemographic characteristics, incarceration was associated with depressive symptoms. More economic disadvantage experiences and depressive symptoms during adolescence were associated with greater frequency of depressive symptoms in young adulthood.

In model 3, stress was associated with depression net of the other coefficients. The effect of incarceration decreased when accounting for stress indicating that incarceration affects mental health partially through stress. Results of a Sobel test for mediation showed that the coefficient for incarceration significantly decreased in magnitude after including stress in the model.

### Results for Self-Reported Poor Health

Table 3 included the unadjusted and multivariable logistic regression of self-reported poor health on criminal justice system involvement, adolescent health risks, stress, and sociodemographic characteristics. The bivariate (unadjusted) analyses indicated that economic disadvantage experiences during adolescence and stress during young adulthood were associated with higher odds of reporting poor health in young adulthood. Model 1 included criminal justice contact and sociodemographic characteristics. Net of sociodemographic controls (race, gender, and age), the association between incarceration and the probability of reporting poor health approached significance. None of the sociodemographic characteristics were significantly associated with self-reported poor health during young adulthood.

In model 2, higher BMI scores during adolescence were not associated with self-reported poor health during young adulthood net of the other variables in the model. The quadratic term continued to be insignificant. In model 3, stress was associated with higher odds of reporting poor health.

To assess whether the effect of arrest only is similar or different to incarceration, we changed the reference group in each model from never arrested to incarcerated (not shown). The effect of arrest was significantly lower than the effect of incarceration on self-reported poor health and depression, and did not differ from the effect of no criminal justice contact. That the arrested only coefficients were not significantly different from the never arrested coefficients demonstrated that any effect on health appears triggered by incarceration.

## DISCUSSION

Incarceration was positively associated with young adults’ self-reported depressive symptoms. Additionally, adolescent health risks (economic disadvantage, earlier depression, problems with drugs) were associated with greater frequency of depressive symptoms. Black compared to White young adults, as well as young adult women, reported more frequent depressive symptoms. Stress mediated the association between incarceration and frequency of depressive symptoms. This finding of mediation supports arguments made by scholars who have posited that the stressfulness of incarceration is likely to overwhelm individuals’ coping abilities and ultimately leave them more depressed than prior to incarceration.^2^ We did not find that incarceration was associated significantly with young adults’ self-reported poor physical health controlling for adolescent health risks and sociodemographic characteristics. Yet, adolescent BMI and stress were associated with young adults’ self-reported poor physical health.

Although studies have begun to explore whether the effects of the criminal justice system extend to early procedures like arrest or police contact, we found no evidence of deleterious effects of arrest for depression (similarly arrest was not significantly associated with poor physical health). Even in supplementary analyses (not shown) which tested experiencing arrest without considering incarceration, there was no significant association between arrest and any health outcomes. It may be the case that being arrested multiple times is damaging to both physical and mental health. An interesting ‘non-finding’ is that adolescent delinquency and problems with drugs were not associated with young adults’ reports of poor health. It is likely that persistent long-term problems with drugs would negatively affect health outcomes.

The results of this study support the need to continue developing theory and research in the area of stress processes and cumulative disadvantage, and how these affect health consequences of incarceration. As demonstrated by our analyses, stress mediated the effects of incarceration experience on depressive symptoms. Importantly, the effect of childhood economic disadvantage on depression remained significant in all of the models suggesting that earlier economic disadvantage has long-term consequences for young adults’ mental health.

We have contributed to the literature on the association between incarceration and health in several ways. Previous studies have suggested that prison conditions negatively impact individuals’ health even after release from prison. Recently, studies established a longitudinal association between juvenile incarceration and long-term health problems and depression.^7^ However, it is plausible that incarceration is not the cause of poor health, but rather reflects selection into poor health by individuals who engaged in earlier health compromising behaviors, which we referred to as adolescent health risks, and who were disadvantaged by their childhood economic standing. Additionally, other scholars hypothesized that the negative effects of incarceration and arrest on health act through the increased stress of the prison environment.^8,9^ Although researchers presume stress is the mechanism through which incarceration affects well-being,^8^ in this paper we tested this relationship.

The arrest and incarceration measures are retrospective. This limitation is potentially problematic because the dependent variables assessed health at the time of the interview. There may be important health consequences for individuals who spent a short period of time in jail and those sentenced to longer duration prison terms. Future data and studies can further address this with a more detailed look at number of arrests, the timing of incarceration experiences, and the duration of incarceration experiences to determine more accurately whether these events triggered increases in stress. Nevertheless, the findings of some differences for depression due to incarceration call attention to their sizable consequences even in the short run. Future research should also focus more so on race/ethnicity differences in incarceration rates. For example, future studies should confirm whether drug offense arrests/incarceration effects on health outcomes differ for White, Black, and Hispanic young adults.^32^ Unfortunately, our data do not permit us to assess this question.

Studies of incarceration and health have often used more in-depth measures of health conditions than the single item self-report poor health measure used in this study. Nevertheless, some scholars have concluded that self-reported overall health is an adequate if not superior way to measure health in survey research. A self-reported measure may be the optimal way to capture health disparities in younger adults because it is highly correlated with objective measures of more serious conditions.^25^ Other studies have focused primarily on older adults or adolescent health. The present study focused on adults who should be in excellent physical health. Thus, finding small effects on health may be indicative of future health problems. Furthermore, additional research is needed on the interplay of the stress indicators. Although they are correlated well enough to combine into a single measure (α=0.83), this does not permit us to elaborate on more specific pathways. We conceptualized this measure of stress as manifestations of proliferating stress via the stress process, however, other pathways are plausible.

The present study combined juvenile and adult criminal justice experience into singular categories that indicated any criminal justice experience. Supplemental analyses of the TARS data did not reveal any significant differences between those who experienced the criminal justice system as juveniles and those who only experienced arrest or incarceration as adults. The present study did not focus on racial disparities in the processes we examined. Researchers have repeatedly shown that racial disparities exist in the experience of economic disadvantage, exposure to incarceration, and a range of health outcomes.^32^ Similarly, with regard to BMI there are differences by race/ethnicity. Supplementary analyses of these data identified some differential processes based on race (results not shown). The findings on stress as a mediating mechanism were not significantly different from the ones presented in the present study while controlling for race. Thus, an exploration of racial patterns would likely be fruitful avenue for a future research project. Our next step is to determine why the interrelationships explored above might be similar or vary based on race/ethnicity. Gender differences in health (e.g., depression) and odds of system exposure also suggest the need to explore similarities and differences in the nature of these pathways. The relatively limited statistical power of the incarcerated female sample prevented a thorough exploration of these differences.

Several of these limitations (relatively young age of the sample, short time window), are likely to bias the study against our results. Thus, this study represents a rather conservative test of the proposed relationships. However, with a great deal of possible unmeasured or unexplored biases, results should be interpreted cautiously. Still, the striking differences between the incarcerated and not incarcerated are worthy of future scholarly consideration.

## PUBLIC HEALTH IMPLICATIONS

Our results highlight the need to assess adolescents’ experiences that increase the probability of criminal justice contact. Findings on self-reported poor health indicated that incarceration is not significantly associated with poor self-reported physical health when accounting for other important factors. Importantly this study may be too early in the lifespan to gauge true deleterious effects on self-reported physical health. In contrast, incarceration influenced depression net of the adolescent lifestyle and disadvantage factors. Although it is likely that physical health and well-being are influenced by multiple factors, and not solely from incarceration, criminal justice agencies have a responsibility and opportunity to provide health programs in prison and support for health education post-release.^33^ Ohio’s leadership has proclaimed a commitment to be a leader in criminal justice reform amidst mass incarceration in the state.^34^ Among the much needed reforms are additional mental and physical healthcare services provided to prisoners. Ohio has committed previously to programs aimed at providing mental health services to ex-offenders.^35^ Given the significant effects on depression found in this study focusing on emotional well-being and coping with stress should make supporting such programs a priority. Supporting young adults’ mental and physical health post-release will increase the likelihood that this period will lead to efficacious actions including gainful activity and reduced odds of continued reliance on ineffective coping strategies such as substance use. Earlier additions to Ohio reentry programs aimed at reducing prison and jail populations are promising particularly for the juvenile justice system,^36^ but further reform will enhance the chances of adult offenders experiencing better mental health after release. Setting our study in Lucas County provides an example of a typical American city, which sets the stage for generalizability beyond the state of Ohio as well.^23^ Ultimately, by addressing these root causes of problems with drugs and other criminal activities, these changes may help to alleviate the rising incarceration rates in Ohio.

## Figures and Tables

**Table 1.** Descriptive Statistics and Group Comparisons for Criminal Justice Involvement

	Full Sample	Criminal Justice Involvement		
	n=990	Never Arrested n=606	Arrested n=260	Incarcerated n=124
	Mean±SD/ Percentage	Mean±SD/ Percentage	Mean±SD/ Percentage	Mean±SD/ Percentage
**Dependent Variables**				
Depressive symptoms (log transformed)	0.73±0.46	0.70±0.47	0.73±0.47	0.97±0.44^ab^
Poor health (%)	11.5%	10.6%	11.6%	17.4%^ab^
**Independent Variables**				
Economic disadvantage				
0 reported experiences	40.0%	47.7%	34.9%^a^	13.3%^ab^
1 reported experience	22.3%	21.6%	21.6%	27.3%^ab^
2 reported experiences	21.2%	18.4%	23.5%^a^	29.7%^ab^
3+ reported experiences	16.5%	12.3%	20.1%^a^	30.7%^ab^
Adolescent Depression	2.30±1.10	2.30±1.14	2.29±1.05	2.38± 1.22
BMI	0.00±5.44	0.01±5.70	−0.24±5.31^a^	0.35±5.67^ab^
Delinquency	0.29±0.51	0.21±0.42	0.40±0.57^a^	0.54±0.70^a^
Drug use	1.12±0.52	1.07±0.31	1.16±0.52^a^	1.29±0.58^a^
**Mediator**				
Stress	2.11±0.71	2.09±0.70	2.06±0.73	2.34±0.80^ab^
**Sociodemographic factors**				
Race/Ethnicity				
White	66.5%	72.1%	63.8%^a^	44.4%^ab^
Black	22.0%	17.7%	25.8%^a^	35.5%^ab^
Hispanic	11.1%	10.1%	10.2%^a^	20.2%^ab^
Age	26.38	26.44±1.86	26.17±1.18	26.36±1.55
Gender				
Male	46.2%	37.5%	58.9	62.1%^a^
Female	53.8%	62.5%	41.1	37.9%^a^

Source: Toledo Adolescent Relationships Study 2000–2012

Dependent variables collected in fifth interview (2011–2012)

Independent variables collected at the first interview (2000–2001)

Criminal justice involvement categories are retrospective from the fifth interview (2011–2012)

**Note**:

a Value is significantly different from the never arrested group at p<.05

b Value is significantly different between arrested and incarcerated groups at p<.05

**Table 2.** Unadjusted and Multivariable OLS Regression of Depressionab on Criminal Justice Involvement, Sociodemographic Characteristics, Child Disadvantage, Adolescent Health Risks and Stress

	Unadjusted		Model 1		Model 2		Model 3	
	b	(SE)	b	(SE)	b	(SE)	b	(SE)
Intercept			0.756***	(0.21)	0.594*	(0.26)	−0.310	(0.24)
**Criminal Justice System Involvement** ^b^								
Arrest (ref=never arrested)	0.032*	(0.02)	0.027	(0.03)	0.016	(0.03)	0.012	(0.03)
Incarceration	0.226***	(0.02)	0.214***	(0.05)	0.166***	(0.05)	0.128*	(0.04)
**Adolescent Health Risk Factors** ^c^								
Economic Disadvantage^d^ (ref=no disadvantage)								
1 reported experience	0.049**	(0.04)			0.015	(0.04)	0.022	(0.03)
2 reported experiences	0.223**	(0.04)			0.143***	(0.04)	0.129***	(0.04)
3+ reported experiences	0.305***	(0.07)			0.233**	(0.08)	0.232**	(0.07)
Depression	0.106***	(0.01)			0.094***	(0.01)	0.063***	(0.01)
BMI	0.008**	(0.00)			−0.011	(0.01)	−0.014	(0.01)
BMI^2^	0.000*	(0.00)			0.000	(0.00)	0.000	(0.00)
Delinquency	0.061*	(0.03)			−0.004	(0.01)	−0.013	(0.01)
Drug problems	0.098	(0.04)			0.053*	(0.04)	0.083*	(0.04)
**Mediator**								
Stress	0.305	(0.02)					0.286***	(0.02)
**Sociodemographic Characteristics** ^c^								
Race/ethnicity (ref=non-Hispanic white)								
Black	0.160***	(0.04)	0.099***	(0.04)	0.086*	(0.04)	0.102*	(0.04)
Hispanic	0.068	(0.05	0.036	(0.05)	−0.010	(0.05)	−0.008	(0.04)
Gender (ref=male)	0.020	(0.03)	0.067	(0.03)	0.016	(0.03)	0.008	(0.03)
Age	0.025	(0.05)	0.001	(0.01)	−0.015	(0.01)	−0.00	(0.01)

Source: Toledo Adolescent Relationship Study (TARS) 2001–2012

Notes:

* p<.05,

** p<.01,

*** p<.001

a Depression is a log transformed mean scale derived from the CESD (Radloff 1979)

b Measured at the fifth interview (2012)

c Measured at the first interview (2001)

d Measured with the parent questionnaire at the first interview (2001)

**Table 3.** Unadjusted and Multivariate Logistic Regression of Poor Health on Criminal Justice Involvement, Sociodemographic Characteristics, Child Disadvantage, Adolescent Health Risks and Stress

	Unadjusted		Model 1		Model 2		Model 3	
	OR	95% CI	OR	95% CI	OR	95% CI	OR	95% CI
**Criminal Justice System Involvement** ^b^								
Arrest (ref=never arrested)	1.11	(0.69, 1.73)	1.14	(0.71, 1.83)	1.14	(0.70, 1.86)	1.15	(0.70, 1.90)
Incarceration	1.67	(0.99, 2.86)	1.69	(0.97, 2.96)	1.49	(0.82, 2.72)	1.33	(0.72, 2.47)
**Adolescent Health Risk Factors** ^c^								
Economic Disadvantage^d^ (ref=no disadvantage)								
1 reported experience	1.63	(0.94, 2.82)			1.49	(0.84, 2.64)	1.49	(0.83, 2.67)
2 reported experiences	2.61	(1.56, 4.37)			2.33	(1.32, 4.10)	2.24	(1.26, 4.00)
3+ reported experiences	3.23	(1.36, 7.64)			3.36	(1.33, 8.50)	3.50	(1.36, 9.04)
BMI	1.08	(1.05, 1.12)			1.06	(0.89, 1.27)	1.05	(0.88, 1.26)
BMI^2^	1.01	(1.01, 1.01)			1.00	(0.99, 1.00)	1.00	(0.99, 1.00)
Delinquency	1.05	(0.72, 1.53)			1.04	(0.86, 1.27)	1.01	(0.83, 1.23)
Drug use	0.92	(0.56, 1.53)			0.70	(0.34, 1.43)	0.71	(0.34, 1.48)
**Mediator**								
Stress	2.17	(1.69, 2.77)					1.96	(1.51, 2.53)
**Sociodemographic Characteristics** ^c^								
Race/ethnicity (ref=white)								
Black	1.44	(0.91, 2.27)	1.34	(0.85, 2.15)	0.89	(0.53, 1.49)	0.849	(0.49, 1.46)
Hispanic	1.06	(0.56, 2.04)	0.99	(0.51, 1.91)	0.69	(0.34, 1.38)	0.671	(0.33, 1.37)
Gender (ref=male)	1.24	(0.84, 1.84)	1.34	(0.89, 2.01)	1.23	(0.80, 1.87)	1.181	(0.77, 1.82)
Age	1.02	(0.92, 1.14)	1.03	(0.92, 1.14)	0.99	(0.88, 1.10)	1.021	(0.91, 1.15)

Notes: Multivariate results for self-reported health using Toledo Adolescent Relationship Study (TARS) 2001–2012

a Better than poor health was treated as the reference group when determining the self-reported health ORs

b Health, criminal justice involvement, and stress measured at the fifth interview (2012)

c Adolescent Health Risks and sociodemogrpahic controls measured at the first interview (2001)

d Adolescent economic disadvantage measured with the parent questionnaire at the first interview (2001)
